# Primary hydatid cyst of the gallbladder: an unusual localization diagnosed by Magnetic Resonance Imaging (MRI)

**DOI:** 10.11604/pamj.2013.14.15.1424

**Published:** 2013-01-09

**Authors:** Rabii Noomene, Anis Ben Maamer, Ahmed Bouhafa, Noomen Haoues, Abdelaziz Oueslati, Abderraouf Cherif

**Affiliations:** 1 Service de chirurgie générale hôpital Habib Thameur, Tunis, Tunisie

**Keywords:** Primary hydatid disease, Gallbladder, Magnetic resonance imaging (MRI), Cholecystectomy

## Abstract

Hydatid disease is endemic in Tunisia and has been considered as one of the most common surgical pathology. Several localizations have been described, but hydatidosis of the liver is the most frequent clinical entity. Primary hydatid cyst of the gallbladder is very rare. We report in this observation a new case of primary hydatid cyst of the gallbladder diagnosed by Magnetic Resonance Imaging (MRI).

## Introduction

Human hydatid disease is a particularly frequent in Tunisia where echinococcosis is endemic. It is a zoonotic infection caused by Echinococcus granulosus for more than 95% of cases while Echinococcus multilocularis is found in fewer than 5% of cases. The main species pathogenic for humans in mediterranean and southern european countries is Echinococcus granulosus. Hydatid cyst of the liver; witch is the most frequent location is easily diagnosed by ultrasound examination. The other abdominal cysts need generally more investigations. CT abdominal scan is usually performed to characterize cystic abdominal masses. For hydatid cyst; it allows the diagnosis in up to 90% of cases. However magnetic resonance imaging (MRI) is rarely needed. Primary hydatid cyst of the gallbladder is an unusual and very rare localization of hydatid disease and must be segregated from gallbladder daughter cysts secondary to liver primary hydatidosis.

The aim of this case report is to highlight the diagnostic features of this rare clinical entity.

## Patient and observation

**Figure 1:**
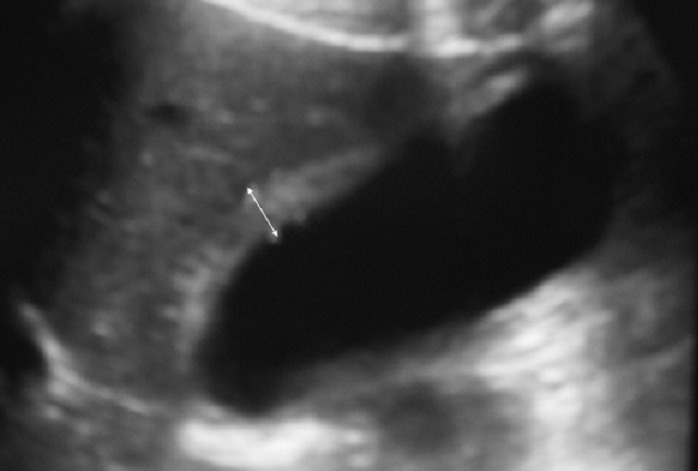
Ultrasound examination showing deformed gallbladder with a localized thickening in its wall

A 32-year-old woman was admitted suffering from constant mild pain in the right hypochondrium reflecting to the epigastrium; and often accompanied by nausea during the previous 6 months. There was no history of fever or jaundice. The physical examination didn't show abnormal abdominal signs except tenderness in the right upper quadrant of the abdomen. Routine blood tests proved unremarkable. Plain abdominal X-rays was normal. The ultrasound examination showed that her gallbladder was deformed with a localised thickening of its wall (Figure 1). There was no image of gallstone.

**Figure 2:**
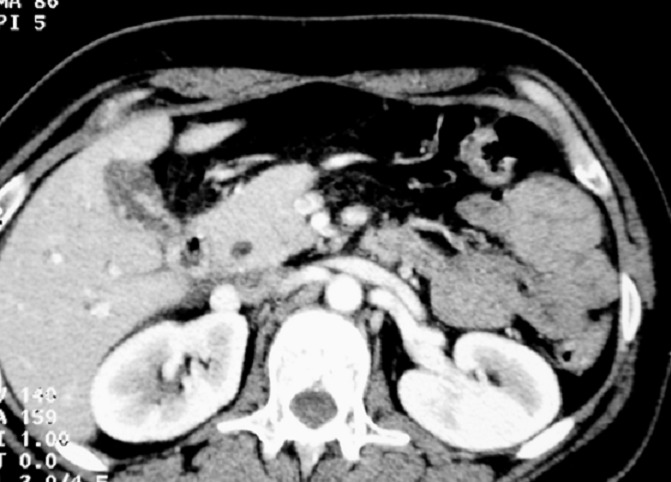
Gallbladder aspect in CT abdominal scan

**Figure 3:**
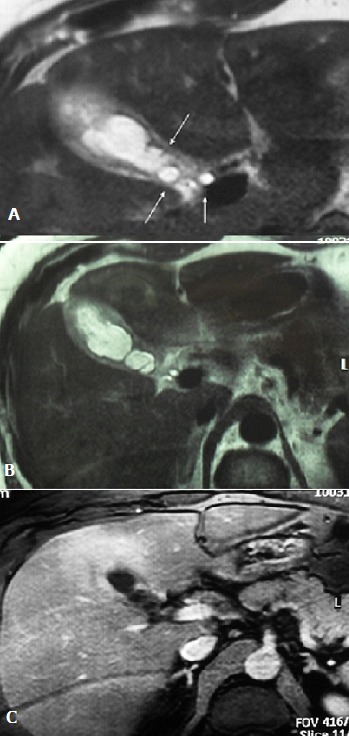
T2-weighted sequence demonstrating daughter cysts in the lumen of gallbladder (A,B); hepatic parenchyma free of cystic lesions (C)

To eliminate gallbladder neoplasm; a CT abdominal scan was performed showing inflammatory gallbladder wall which is free of suspect lesions (Figure 2). Against this diagnosis difficulty; it has been decided to practice a magnetic resonance abdominal imaging. This examination showed many daughter cysts in the lumen of the gallbladder (Figure 3-A, B); while hepatic parenchyma proved absolutely normal (Figure 3-C)

**Figure 4:**
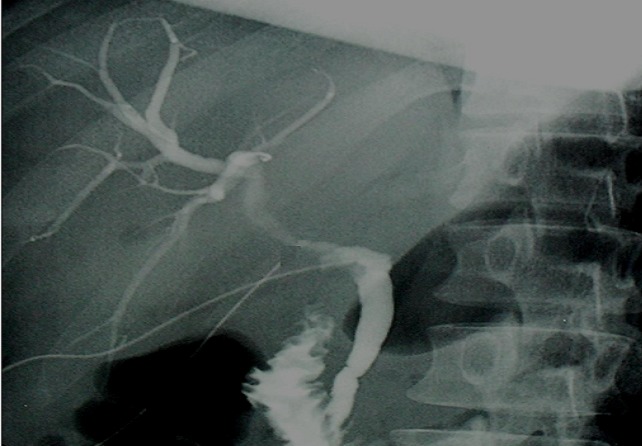
Preoperative cholangiographie

**Figure 5:**
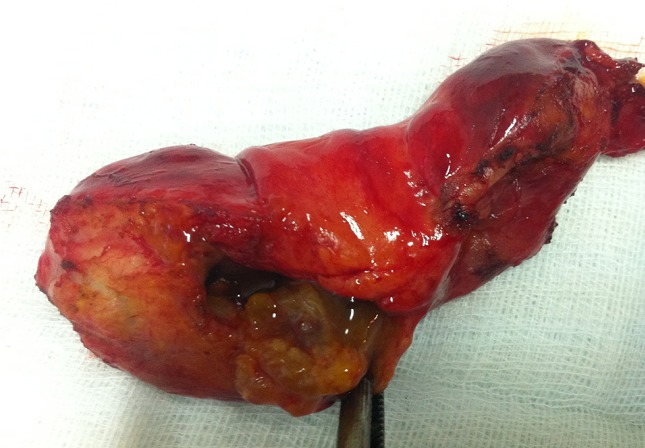
Macroscopic examination showing daughter cysts showing normal aspect of common bile duct in the lumen of the gallbladder

Antiechinococcal antibodies were not found in serum. The diagnosis of primary hydatid cyst of gallbladder was supported and surgery has been decided. The patient underwent right subcostal laparotomy. Intraoperatively it has been found a cyst of the body of the gallbladder which is inflammatory and deformed. No other cysts were found in the liver and peritoneal cavity. A cholecystectomy was performed. It permitted a total removal of the cyst without rupture. A preoperative cholangiography searching daughter cysts in the common bile duct proved unremarkable (Figure 4). The patient's postoperative course was uneventful and she was discharged in the fourth postoperative day. The histopathology confirmed the presence of hydatid cyst of the gallbladder (Figure 5). At six month follow up, the patient has had no recurrence of hydatid disease.

## Discussion

Hydatidosis is a human disease caused by the larval form of Taenia echinococcus. This parasite is endemic in many sheep- and cattle-raising countries [1, 2]. Hydatid disease is still a major health problem that affects both humans and animals in Tunisia. The average annual incidence rate is 11. 3 per 100,000 inhabitants [2]. The vast majority of the patients reside in rural areas. Echinococcus lives in the gut of the dog and wild canines that represents the definitive host. Humans are accidentally infected by the orofecal route [3, 4]. The larvae may come to rest and develop into hydatid cysts in any part of the body, but the liver (70%) and lungs (20%) are most commonly affected and are considered as the first and the second filter to the embryos circulation. In fact the embryos pass through the intestinal wall and into portal system to the liver. Via the hepatic veins, inferior vena cava and heart; embryos may pass through the liver and lungs to spread throughout the body [4]. Primary hydatid cyst of the gallbladder is a very rare entity. Cysts can be located into the lumen of the gallbladder or on its external surface [4, 5]. The pathogenesis of the primary gallbladder hydatid cysts is not well-documented. Besides the spread of echinococcal embryos through the portal circulation, other routes of spread may exist such as biliary, lymphatic or peritoneal routes [5, 6]. Many hypotheses are proposed to explain the exact pathogenesis of the primary gallbladder hydatid cysts. One of the first supported is contamination of gallbladder by biliary route; but that seems to be inappropriate for the external surface located cysts and often require a previous contamination of the liver. Spread of echinococcal embryos through the lymphatic circulation after intestinal absorption is possible and may explain the lumen's gallbladder cysts [6]. Other modalities can be discussed such as contamination of the gallbladder wall after insufficiently protected surgery of hepatic hydatid cysts [6, 7]. Clinical presentation consists generally in pain localised in the right hypochondrium and rarely in the epigastrium [7]. Jaundice may occur by compression of the common bile duct by a big cyst of the gallbladder wall or after migration of daughter cysts into the biliary tract; but we didn't find publications describing those complications of gallbladder cyst.

Those manifestations lead habitually to ultrasound examination which is the most important investigation. It supports the diagnosis in up to 90% of cases in hepatic hydrated cysts. This examination can divide hydrated cysts into five groups depending on age of cyst. This classification; established by Garb et al; is always used in the Tunisian literature since 1981^7^, ^8^]. Floating undulating membranes, multiple septa within a calcified cyst are specific features for hydrated cyst and seem to be very helpful for the diagnosis. However for unusual localisations; ultrasound may be useful but with a very lower sensibility rate. In such cases computed tomography scan is often required. This examination is very useful to make a differential diagnosis from other cysts, such as choledochal cysts, pancreatic pseudocysts, and cystic neoplasms [8, 9]. Only new generations of CT scan making 3D reconstructions seems to be able to sow specific images of daughter cyst. In Tunisia the endemic criteria is usually considered as an important element of positive diagnosis. Magnetic resonance imaging is rarely required in the investigation of hydatidosis and it is reserved for difficult diagnosis and some unusual location such as pancreatic or pericardial cysts [9]. Our observation demonstrates the ability of current MR techniques to provide clear images of hydatid cysts. T2-weighted sequence demonstrates daughter cysts most clearly due to the high contrast resolution between highsignal intensity of the intraluminal fluid and the relatively low signal intensity of cystic wall. This examination provides a better analysis of hepatic parenchyma and can separate primary gallbladder cysts from those secondary to liver contamination. Sometimes serological tests can help to delineate the cyst's nature in the case of non specific imaging finding [9].

Surgery is always required for the treatment [9, 10]. According to the Tunisian experience; there is no place for medical treatment by Albendazol which is indicated only for non operable patients or after surgery of multiple intrabdominal hydatid cysts. Laparoscopic surgery is inappropriate for the treatment because the risk of spillage of the cyst content and dissemination of hydatid disease. Patients should undergo right subcostal laparotomy witch permit a total exploration of the right hypocondrium and an efficient protection of peritoneal cavity. Two choices are offered in the surgical management of hydatid cysts. Total pericystectomy witch permit a radical removal of the cyst and a low rate of recurrence. However it is not always possible and it expose to the risk of preoperative haemorrhage. Te second choice is the partial pericystectomy witch is safe and efficient; but it expose to recurrence [10]. The localisation of the hydatid cyst in the gallbladder seems to offer the possibility of total removal in all cases. Successful total pericystectomy was performed in all reported observations. Cholecytectomy is sufficient for cysts involving into the lumen. For the external surface located cysts total percystectomy may be performed sometimes with a partial removal of hepatic tissue [9, 10].

## Conclusion

Primary hydatid cyst of the gallbladder is a very rare clinical entity. Preoperative diagnosis seems to be possible with a specific imaging finding which can be offered by CT scan or RMN such as in our observation. The gallbladder primary hydatid cyst may have different spread routs of parasite embryos. Prognosis is better than hepatic localisations due to earlier diagnosis and the possibility and the safety of total pericystectomy.
